# WHO 2022 updates on follicular cell and c-cell derived thyroid neoplasm

**DOI:** 10.25122/jml-2023-0270

**Published:** 2024-01

**Authors:** Parth Goswami, Tarang Patel, Rushang Dave, Gyanendra Singh, Anurag Singh, Tushar Kalonia

**Affiliations:** 1 Department of Pathology, All India Institute of Medical Sciences, Rajkot, Gurjat, India; 2 King George's Medical University, Lucknow, India; 3 Department of Pathology, Sharda Hospital, Greater Noida, India

**Keywords:** Thyroid neoplasm, Follicular cell neoplasm, C-cell neoplasm

## Abstract

The latest edition of the WHO Classification of thyroid tumors was released in 2022 and incorporates novel concepts vital to patient management. Thyroid follicular nodular disease is a term used to collectively represent a wide variety of benign and non-neoplastic lesions, including both clonal and non-clonal proliferations that manifest clinically as multinodular goiter. Thyroid neoplasms develop from follicular cells and can be either benign, low-risk, or malignant. To avoid classifying all lesions under 1 cm in diameter as low-risk illnesses, the new classification method highlights the need for subtyping papillary thyroid cancer based on histomorphologic indicators rather than tumor size. Formerly known as the cribriform-morular variety of papillary thyroid carcinoma, this tumor is now more commonly referred to by its more accurate name, cribriform-morular thyroid carcinoma. Its histogenesis is unknown. Similar to the traditional definition of ‘poorly differentiated thyroid carcinoma’ according to the Turin criteria, the newly defined ‘differentiated high-grade thyroid carcinoma’ encompasses papillary thyroid cancer, follicular thyroid carcinomas, and oncocytic carcinomas with high-grade characteristics linked to worse prognosis. The squamous cell subtype of anaplastic thyroid cancer has also recently been characterized as a distinct morphologic pattern. In this article, we will discuss the latest revision to the World Health Organization's classification system for thyroid cancer.

## INTRODUCTION

The fifth edition of the World Health Organization (WHO) Classification of Endocrine and Neuroendocrine Tumors is a timely publication that easily lends itself to years of experience in the field of diagnostic and molecular thyroid pathology. It contains appropriate changes to nomenclature, grading, and prognostication of thyroid proliferations based on pathologic features and molecular profile. Therefore, endocrinologists and practicing physicians who manage thyroid nodules should acquaint themselves with this new classification scheme [1].

The thyroid gland is the source of the most common endocrine tumors. The clinical entity known as multinodular goiter has been used for pathology diagnosis. However, this is inappropriate and pathologists use the terminology such as hyperplasia, adenomatous, and adenomatoid for such lesion. Clonal events, such as gene mutations, may be the deciding element in explaining why a few multinodular goiters develop into malignancies.

An alternative terminology proposed to address this is ‘thyroid follicular nodular disease’, a term that avoids defining a lesion as hyperplastic, neoplastic, or the contradictory ‘adenomatous hyperplasia’ [2]. According to the most recent classification, follicular adenoma with papillary architecture is included as a new entity, as is shown in table 1 [1].

**Table 1:** Major revisions to the classification and nomenclature of thyroid tumors

Variable	2022 WHO classification	2017 WHO classification
**Terms**	Oncocytic cell	Hürthle cell
	Subtype	Variant
**Mitotic count**	Number of mitoses per 2 mm^2^	Number of mitoses per ten high power fields
**Gene fusions (separator between gene symbols)**	Double colon (::)	Hyphen (-) or forward slash (/)
**Tumor type/subtype**	Thyroid follicular nodular disease: new term to describe multifocal benign follicular nodules presenting as a multinodular goiter	Not applicable
	Follicular adenoma with papillary architecture: a separate type	Hyperfunctioning adenoma, follicular adenoma with papillary hyperplasia: variants of FA
	Subcentimeter NIFTP, oncocytic NIFTP: new subtypes of NIFTP	Not applicable
	Invasive encapsulated follicular variant papillary carcinoma: separated from other subtypes of papillary thyroid cancer (PTC)	Invasive encapsulated follicular variant of PTC
	None, specific histologic subtyping is needed	Papillary microcarcinoma variant
	Oncocytic adenoma of the thyroid	Hürthle cell adenoma
	Oncocytic carcinoma of the thyroid	Hürthle cell carcinoma
	Follicular-derived carcinomas, high-grade	Not applicable
	Differentiated high-grade thyroid carcinoma	Differentiated thyroid carcinoma (PTC, FTC, or oncocytic carcinoma of the thyroid (OCA)) with high-grade features
	Cribriform-morular thyroid carcinoma: separated from PTC	Cribriform-morular variant of PTC
	Anaplastic thyroid carcinoma, squamous cell carcinoma pattern	Squamous cell carcinoma
	Thyroblastoma	Malignant teratoma

Molecular analyses of individual nodules in such cases have revealed that a good proportion of goitrous nodules is monoclonal and represent neoplastic proliferations, making it impossible to distinguish between non-neoplastic and benign neoplastic follicular neoplasms, i.e., adenomas based on morphology alone [3].

Low-risk neoplasms, such as non-invasive follicular thyroid neoplasm with papillary like nuclear features (NIFTP), tumors of uncertain malignant potential (UMP), and hyalinizing trabecular tumor (HTT) are included in the classification, as they behave neutrally. They are benign-malignant tumors. However, metastasis is unlikely. Tumor nomenclature reduces wasteful treatment of low-risk neoplasms [4].

Hürthle cell carcinoma is replaced by the term ‘Oncocytic carcinoma’. In the follicular thyroid carcinoma (FTC), oncocytic carcinoma is defined as a population of oncocytic cells that exceeds 75%.

The 5^th^ edition of thyroid tumor classification proposed by WHO identifies two groups of follicular cell–derived high-grade non-anaplastic carcinomas with an intermediate prognostic risk (Supplementary Table 1).

**Supplementary Table 1:** WHO classification of thyroid neoplasms, 5^th^ edition

**Developmental abnormalities**
1. Thyroglossal duct cyst
2. Other congenital thyroid abnormalities
**Follicular cell–derived neoplasms**
1. Benign tumors
a. Thyroid follicular nodular disease
b. Follicular adenoma
c. Follicular adenoma with papillary architecture
d. Oncocytic adenoma of the thyroid
**2. Low-risk neoplasms**
a. Non-invasive follicular thyroid neoplasm with papillary-like nuclear features
b. Thyroid tumors of uncertain malignant potential
c. Hyalinizing trabecular tumor
**3. Malignant neoplasms**
a. Follicular thyroid carcinoma
b. Invasive encapsulated follicular variant papillary carcinoma
c. Papillary thyroid carcinoma
d. Oncocytic carcinoma of the thyroid
e. Follicular-derived carcinomas, high-grade
i. Differentiated high-grade thyroid carcinoma
ii. Poorly differentiated thyroid carcinoma
f. Anaplastic follicular cell–derived thyroid carcinoma
Thyroid C-cell–derived carcinoma
1. Medullary thyroid carcinoma
Mixed medullary and follicular cell–derived carcinomas
Salivary gland–type carcinomas of the thyroid
1. Mucoepidermoid carcinoma of the thyroid
2. Secretory carcinoma of salivary gland type
Thyroid tumors of uncertain histogenesis
1. Sclerosing mucoepidermoid carcinoma with eosinophilia
2. Cribriform morular thyroid carcinoma
Thymic tumors within the thyroid
1. Thymoma family
2. Spindle epithelial tumor with thymus-like elements
3. Thymic carcinoma family
Embryonal thyroid neoplasms
1. Thyroblastoma

## Follicular cell-derived neoplasms

### Benign tumors

**Figure 1:**
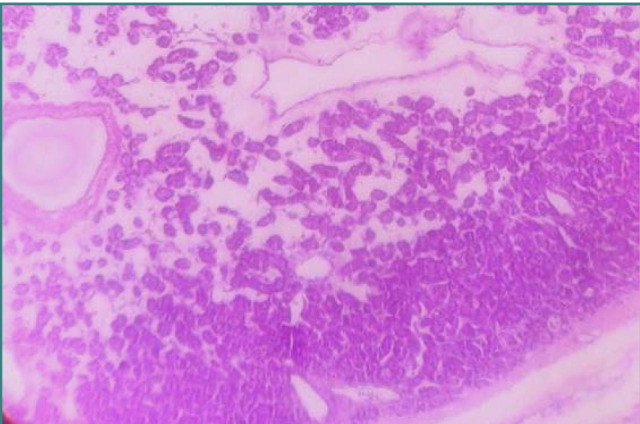
Hematoxylin&Eosin-stained section of Follicular adenoma of the thyroid. The tumor cells are arranged in a trabecular and microfollicular pattern (200X).

In the WHO 4^th^ thyroid classification, only one benign thyroid pathology was recognized, namely follicular adenoma [5] (Figure 1). To better reflect the clonal and non-clonal proliferations that clinically show as multinodular goiter, the diverse collection of non-neoplastic and benign neoplastic lesions is now referred to as 'thyroid follicular nodular disease [6-10]. Histology cannot distinguish between neoplastic and hyperplastic lesions. To solve this problem, a new classification emerged, encompassing a new term, respectively follicular nodular disease. Criteria for follicular nodular disease are similar to the histologic findings of nodular hyperplasia. Thyroid pathologies like adenomatous nodules, adenomatous hyperplasia, nodular hyperplasia, and multinodular goiter are also allowed as per the WHO guideline [2].

Some follicular adenomas have a papillary architecture within the follicle. They lack the nuclear features of papillary thyroid carcinoma and are hyperfunctioning. They are known as toxic adenomas or hyperfunctioning adenomas, although the previous classification included them in follicular adenomas. According to the most recent classification, follicular adenoma with papillary architecture is included as a new entity.

Follicular adenomas with papillary architecture do not have the RAS mutation seen in follicular adenomas. However, they have mutations in the *GNAS* and *TSHR* genes [11]. Research shows that mutations are responsible for unrestricted stimulation and overfunction [12].

**Figure 2:**
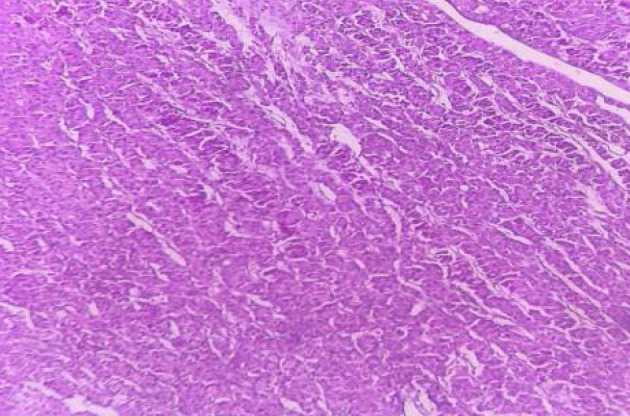
Hematoxylin&Eosin-stained section of oncocytic adenoma of the thyroid. The tumor cells are arranged in a trabecular and solid pattern with cells having large size, deeply eosinophilic and granular cytoplasm, a large nucleus with prominent nucleolus (100X).

In 1894, Karl Hürthle named the parafollicular cells of the thyroid Hürthle cells. This terminology changes to ‘oncocytic cells’ in this classification. They are follicular cells with abundant eosinophilic granular cytoplasm caused by an excess of mitochondria (Figure 2). Moreover, follicular adenoma also shows oncocytic change. To qualify as an oncocytic adenoma, more than 75% of oncocytic cells are required.

### Low-risk neoplasms

In the recent classification, a new category of low-risk neoplasm was added. Their behavior is neither benign nor malignant, but intermediate between these two forms. They have the potential to metastasize, but chances are low. NIFTP, UMP, and HTT are all included in this category. The goal of the tumor terminology is to decrease the possibility of unnecessary treatment of low-risk neoplasms [4].

**Figure 3:**
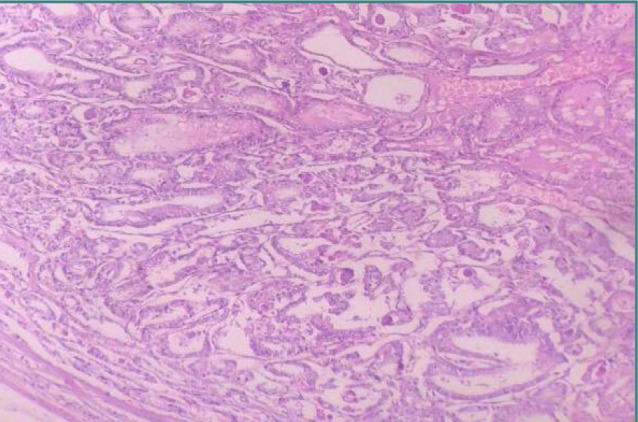
Hematoxylin & Eosin stained section of NIFTP. The tumor cells are arranged in a follicular pattern with cells having nuclear features of papillary thyroid carcinoma (200X).

In 2016, an international team of specialists defined NIFTP terminology in thyroid neoplasms. NIFTP lesions must be encapsulated with nuclear features of papillary neoplasms to be diagnosed (Figure 3). Nuclear features of papillary tumors are nuclear enlargement, nuclear membrane irregularity, overlapping, and grooves. For each class of nuclear characteristics, scores from zero to three are given.

Psammoma bodies seen in papillary thyroid cancer (PTC) are not present in NIFTP. FTC has angioinvasion and capsular invasion, but NIFTP does not. NIFTP excludes more than 1% of the papillae [13]. NIFTP has been added in the fourth edition of the WHO endocrine tumors, meeting all of these diagnostic criteria.

Often, in NIFTP patients with less than 1% of papillae, the *BRAF v600E* mutation seen. Moreover, lymph node metastases were also observed in many cases and the criteria were changed by the NIFTP consensus group in 2018 [14-17]. However, in subsequent studies, in NIFTP patients with less than 1% of true papillae, no metastasis was found [18]. Therefore, the WHO 2022 classification is continuous with less than 1% true papillae.

Previously, the NIFTP category, excluded oncocytic tumors less than 1 cm in size that had similar histologic criteria to NIFTP, as they were. diagnosed as a subtype of PTC. In the recent classification, they are considered subtypes of NIFTP. To qualify as oncocytic NIFTP 75% of cells must have an oncocytic morphology [19]. Subcentimetric NIFTP has a size range of less than 1 cm and greater than 2 mm [20].

Due to the presence of some overlapping histological features, sometimes distinguishing between benign or malignant follicular thyroid tumors is difficult. The phrase ‘TT-UMP’ refers to a category of borderline thyroid tumors. Tumors in this category are encapsulated, follicular patterned, and have architectural and/or cytological criteria that are worrisome but do not meet all of the criteria for malignancy. UMP is classified, in the 2022 classification, into two subtypes, based on nuclear alterations, namely follicular tumor - UMP (FT-UMP) and well-differentiated tumors - UMP (WDT-UMP). FT-UMP lacks PTC's nuclear characteristics, whereas WDT UMP has PTC's nuclear characteristics more prominently displayed. Angioinvasion and capsular infiltration are not present in follicular adenoma or NIFTP, but are present in UMP. There is a similarity in molecular characteristics between NIFTP, UMP, and other follicular tumors. As a result, molecular testing to diagnose preoperative cytology specimens plays no role. Western countries have a higher prevalence (15-20%) of NIFTP compared to Asian countries (0.5–5%) [21].

Lobectomy is the therapy for NIFTP and HTT. Because NIFT and HTT are benign tumours, lobectomy is the preferred treatment method, with no additional radiotherapy required. Because the biological potential of UMP tumours is unknown, constant monitoring is essential.

A hyalinizing trabecular tumor is a rare neoplasm characterized by a trabecular pattern associated with conspicuous intratrabecular hyaline material. An active basal membrane protein produces intratrabecular hyaline material. The diagnosis of this tumor is always challenging because of the overlapping features with papillary and medullary thyroid cancers.

However, significant stromal hyaline material, particularly intratrabecular hyalinization, is relatively uncommon in PTC and NIFTP. PTC would be suggested by the presence of a papillary pattern, scanty, bubble gum-like colloid, psammoma bodies, multiple nuclear grooves, and pseudoinclusions. The use of immunohistochemistry like the presence of membrane and cytoplasmic MIB1 positivity and the absence of widespread HBME-1 and galectin3 staining support HTT. According to recent molecular investigations, *GLIS* gene configurations have been reported in HTT [22,23].

### Follicular thyroid carcinoma and follicular variant of papillary thyroid carcinoma

FVPTC and FTC were almost identical, identifiable from their different nuclear morphology. FVPTC is a follicular tumor with nuclear characteristics similar to PTC. FVPTC has a poorer prognosis than FTC and more lymph node metastases. In the FTC, oncocytic carcinoma is defined as a population of oncocytic cells that exceeds 75%. The prognosis of both tumor types is determined by the extent of invasion. Therefore, the prognosis of tumors that are mildly, angioinvasively, or widely invasive is different.

Different FTCs have different disease-free survival rates after 40 months, namely 45% for widely invasive FTCs, and97% and 81% for minimally and angioinvasive FTCs, respectively [24,25].

The criteria as well as the number of vessels are important in the diagnosis of angioinvasive FTC or FVPTC. However, in a difficult case, the presence of a single focus of vascular invasion combined with the absence of widely invasive growth is sufficient to diagnose angioinvasive FTC or FVPTC.

A total thyroidectomy with neoadjuvant therapy is the treatment of choice for a widely invasive or angioinvasive tumor. In minimally invasive FTC, local resection alone is sufficient.

The 5^th^ edition of thyroid tumor classification proposed by WHO identifies two groups of follicular cell-derived high-grade non-anaplastic carcinomas with a prognostic risk of intermediate category.

### Poorly differentiated thyroid carcinoma and differentiated high-grade thyroid carcinoma

Poorly differentiated thyroid carcinoma (PDTC) and differentiated high-grade thyroid carcinoma (DHGTC) have different developmental patterns solid, insular, and trabecular. These carcinomas are high-grade and derived from cells of follicular origin with minimal histological differentiation (or combinations of these PDTC are behaviorally and morphologically intermediate between well-differentiated and undifferentiated anaplastic carcinomas. In Turin, Italy, a consensus meeting reached an agreement on the diagnostic criteria for PDTC. These criteria include (1) the existence of a solid/trabecular/insular growth pattern, (2) the absence of the usual nuclear characteristics of papillary carcinoma, and (3) the presence of at least one of the following features: twisted nuclei, mitotic activity, 3/10 hpf, and tumor necrosis. For practical utility in the diagnosis of this tumor, an algorithmic technique was developed [26].

**Figure 4:**
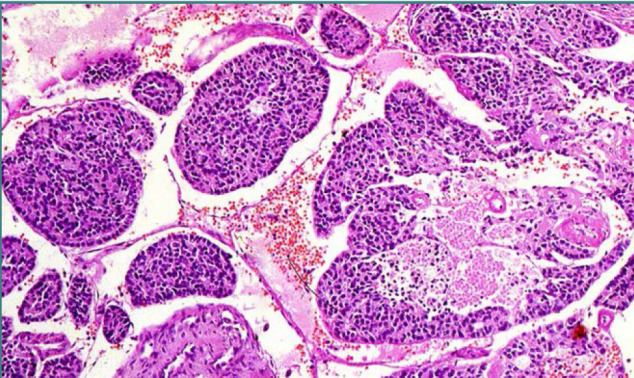
Hematoxylin & Eosin stained section of poorly differentiated carcinoma of the thyroid. The tumor cells are arranged in solid nests, without necrosis and papillary-like nuclear features (400x).

The development pattern of PDTC is insular, solid and/or trabecular. Tumor cells in some cases exhibit small nuclei with clumped dark chromatin with a resinoid appearance, similar to the nuclei of tumor cells of papillary thyroid carcinoma. Necrosis with the presence of necrotic tumor cells and dust of nuclear material is a defining feature of PDTC (Figure 4). If tumor necrosis does not exist, the mitotic count must be at least three mitotic figures/10 hpf/ 2-mm^2^ to be defined as poorly differentiated.

The oncocytic carcinomas which are mitotically active, frequently exhibit necrosis, because they have growth patterns of solid or trabecular type and simulate the criteria for PDTC [26,27,28].

**Figure 5:**
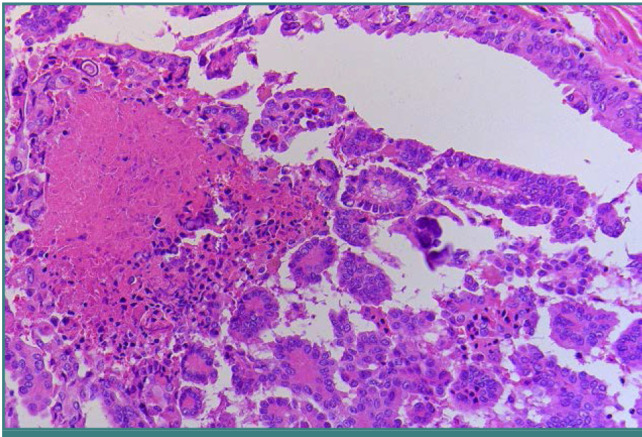
Hematoxylin & Eosin section of HGPTC, with foci of necrosis (400X)

In the great majority of instances, DHGTC exhibits a development pattern that is papillary and comparable to well-differentiated tumors. Although some regions of the tumor may exhibit nuclear expansion and pleomorphism, nuclear characteristics typical of papillary thyroid carcinoma may be present across the entire tumor. Necrosis and/or excessive mitotic activity (~five mitotic figures/10 hpf/2 mm^2^, 400) are the distinctive histologic features that support the diagnosis (Figure 5). There are frequently detected blood vessels, nerve, and lymphatic involvement along with invasion beyond the thyroid [29,30,31].

The WHO encourages the use of the mm^2^ unit of measurement, which is intended to be a standard measure that is not dependent on the type of microscope that is being used and can be applied to digital images of entire slides. The new WHO classification methods do not permit the use of high-power fields because the characteristics of these fields change depending on the type of microscope and ocular that is being applied [32].

Both PDTC and DHGTC exhibit positive immunohistochemical staining for Thyroid transcription factor (TTF1), *PAX8*, cytokeratins (often CK-7), and thyroglobulin (TG). TG typically has modest, localized reactivity that looks like dots. The Ki67 proliferation index is elevated, typically between 10% and 30% [5,33].

According to molecular biology, PDTC and DHGTC have *BRAF V600E* or *RAS* mutation.

The PDTC and DHGTC also have severe secondary mutations, usually in the *TERT* promoter but occasionally in *PIK3CA* and *TP53. RAS* mutations are more prevalent in poorly differentiated thyroid carcinomas as a result of their tight criteria, which calls for the absence of nuclear features of papillary thyroid carcinoma. Contrarily, most of the DHGT are driven by *BRAF V600E* because they primarily exhibit papillary carcinoma-like cytoarchitecture. This explains why DHGTC has a higher tendency to develop cervical lymph node metastases.

The disease-specific survival rate for follicular cell-derived high-grade nonanaplastic carcinomas, not meeting the Turin proposed criteria is similar (56% at 10 years) [33,34,35]. Several studies found that after 10 years, the overall survival percentage for patients treated in PDTCs following the Turin plan was 46%, whereas the survival rate for patients receiving disease-specific treatment was 60% [30,36].

### Anaplastic carcinoma of the thyroid

In the 4^th^ WHO classification of thyroid tumor, the squamous cell carcinoma of the thyroid, which comprises exclusively squamous epithelial cells without any component of differentiated thyroid carcinoma, was considered as an entity separate from the anaplastic carcinoma of the thyroid. There is a lot of proof that this tumor has the morphology of anaplastic thyroid cancer.

In many previous investigations, 87% of instances of squamous cell carcinoma of the thyroid, whether it contained differentiated thyroid cancer or not, had *BRAF V600E* mutations, and its prognosis was comparable to that of anaplastic thyroid carcinoma. Corroborating their follicular cell origin, these squamous cell carcinomas on immunohistochemistry show immunoreactivity for *PAX-8* and *TTF-1* in 91% and 38% of cases respectively [37].

Around 60% of instances of pure squamous cell carcinoma, which meets the 2017 WHO diagnostic criteria of squamous cell carcinoma, carry *BRAF-V600E* mutations, and their prognosis is the same as that of anaplastic carcinoma in general [38].

**Figure 6:**
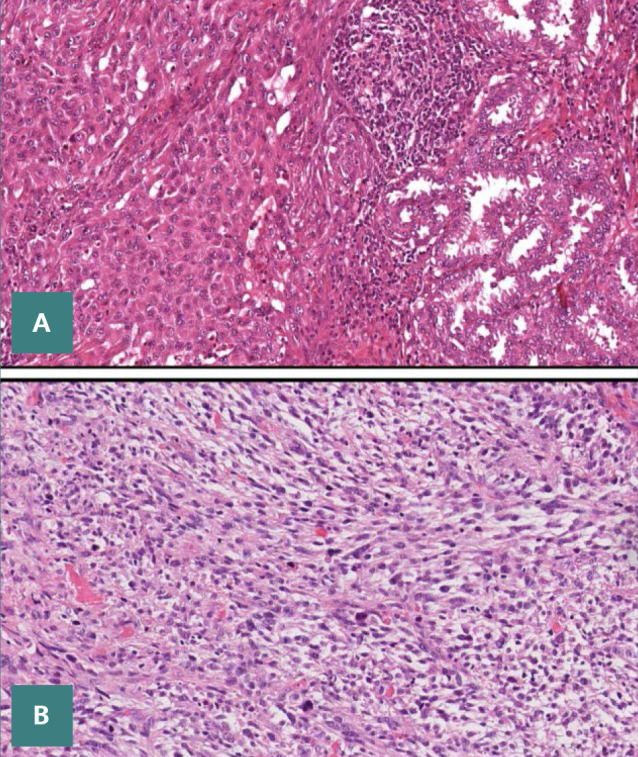
Thyroid cancer that has strong squamous differentiation. (A)The hobnail subtype of papillary thyroid cancer is present in conjunction with the tumor, (B) Anaplastic thyroid cancer with spindle-shaped cells (400X).

Pure squamous cell carcinoma that originated from the thyroid is now categorized as an anaplastic thyroid carcinoma morphologic pattern for the aforementioned reasons (Figure 6).

Consider it necessary to examine these two molecular pathways for anaplastic thyroid carcinoma because BRAF and MEK inhibitors have been shown beneficial in treating anaplastic thyroid carcinoma in numerous prior clinical trials [39].

### Medullary thyroid carcinoma

**Figure 7:**
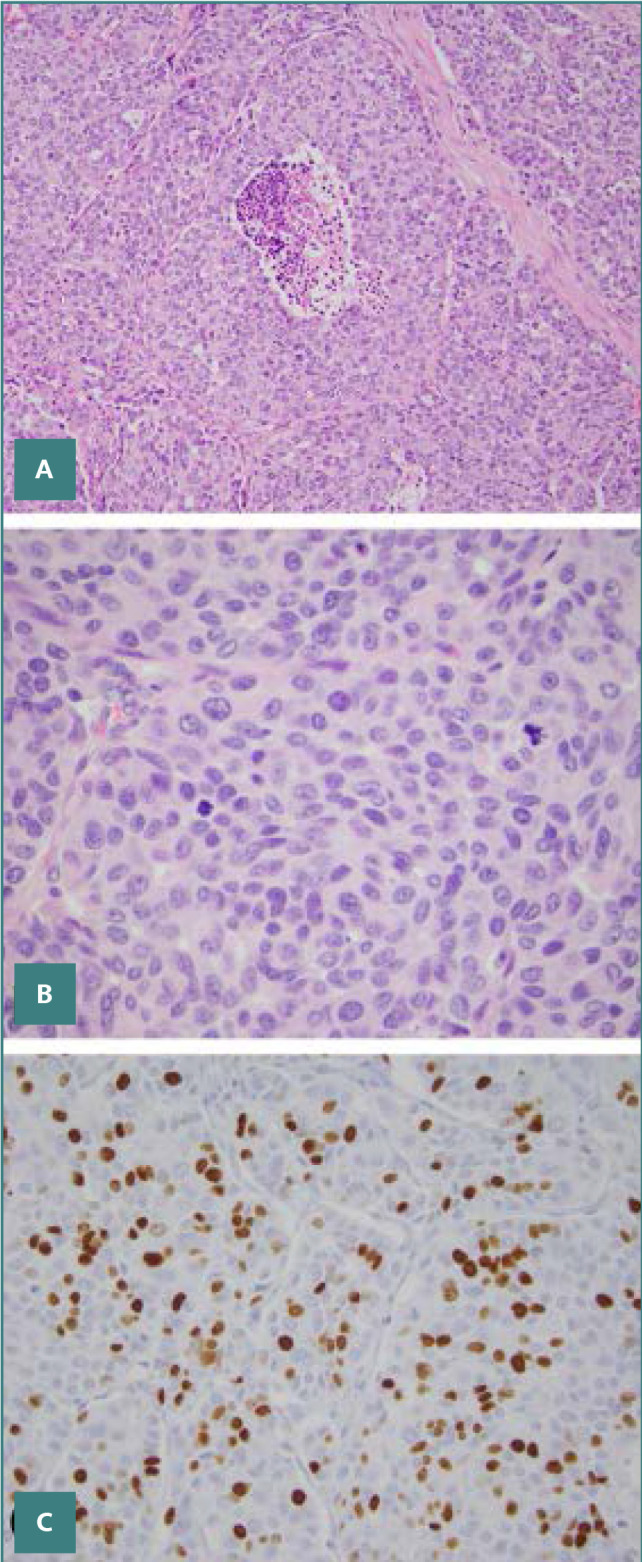
Hematoxylin & Eosin stained section of High-grade medullary thyroid cancer illustration. It has tumor necrosis (A). The mitotic count was 16 per 10 high-power fields (less than 2 mm^2^), and there were clearly visible mitoses present. Ki67 index ~ 35% (B), and (C).

The addition of a grading system is a significant update for medullary carcinoma of the thyroid in the 5^th^ WHO classification of thyroid tumor (Figure 7). There has not been a commonly accepted histological grading system for medullary thyroid carcinoma since its histological characteristics have been described.

The international medullary thyroid cancer grading scheme was designed for the two-tiered grading of medullary thyroid carcinoma, and it was independent of the American Joint Committee on Cancer staging, post-operative serum calcitonin, CEA level, demographic characteristics, and tumor size and margins.

High-grade tumors in this approach are those that exhibit at least one of the three characteristics listed below: tumor necrosis, presence of mitosis five per 2 mm^2^, and/or a Ki67 proliferation index of 5% [40].

Grading of biopsies is therefore not advised because tumor necrosis can be localized, making it necessary to thoroughly examine these tumors.

### Papillary thyroid carcinoma

To eliminate any potential for misunderstanding with the use of the phrase ‘genetic variant(s)’ in molecular diagnostics, the term ‘variant’ has been replaced with ‘subtype’ in the new WHO classification.

Tall cell, columnar cell, and hobnail cell PTC have aggressive clinicopathologic characteristics compared to conventional PTC [41,42].

The new edition (5^th^) of thyroid neoplasms by the WHO emphasizes more papillary thyroid carcinoma subtypes. Diffuse sclerosing is defined by areas of sclerosis, significant lymphatic infiltration, a high number of psammoma bodies, along corresponding chronic lymphocytic thyroiditis [43,44].

Less aggressive PTC subtypes include the solid/trabecular, oncocytic classic, Warthin-like, and clear cell subtypes.

The fifth edition of thyroid tumor classification recommended by WHO included two less frequent subtypes of papillary thyroid carcinoma, the spindle cell PTC and PTC with fibromatosis/desmoid-type/fasciitis-like stroma. Without the use of the appropriate auxiliary techniques, the former can be challenging to identify from other neoplasms. Papillary thyroid carcinoma with associated *BRAF*-mutation embedded in an area of fibromatosis which harbors *CTNNB1* mutation at the molecular level and nuclear localization of β-catenin makes up the latter tumor, which is remarkable in that it has two distinct components [45,46].

PTCs size <1.0 cm have historically been called papillary microcarcinoma. The majority of instances of these tiny PTCs, when identified incidentally, have a favorable prognosis, as is well-documented in the literature. However, a few of these cancers do exhibit aggressive pathologic traits and clinical behaviors, such as metastasis to local and distant sites, and also show recurrence following surgical treatment. Based on this it was recommended that ‘PTC-microcarcinoma’ should not be considered a distinct subtype in the 5^th^ WHO edition of the classification of thyroid tumors [47].

### Invasive encapsulated follicular variant of papillary thyroid carcinoma

The invasive encapsulated follicular variant of papillary thyroid carcinoma (Invasive EFVPTC) is a different entity from its counterpart NIFTP from a prognostic perspective (noninvasive follicular thyroid neoplasm with papillary-like nuclear features). It has the same nuclear characteristics and follicular architecture as NIFTP, but there is an invasion of either the vascular system or the tumor capsule. Women are more likely to be affected than men. (F:M ratio 3.61:1). The disease most frequently manifests in the fourth decade of life [48].

Radiation exposure is thought to play a role in the development of thyroid cancer [49]. There is some evidence that follicular forms of papillary thyroid cancer are linked to increased serum thyroglobulin levels [50]. The prognosis is good, according to a retrospective, multinational, multidisciplinary research of 101 patients with invasive EFVPTC. During a median follow-up of 13 years, only 12 individuals encountered an adverse event, with five patients acquiring distant metastases and two patients dying from the disease [51].

EFVPTC has solid nodules that are well-circumscribed and enclosed; capsular invasion may or may not be visibly apparent [52,53].

The tumor cells are immunoreactive for *TTF1, PAX8*, thyroglobulin, CD19, and Gelectin [54]. It has molecular similarities to follicular adenoma and follicular cancer (RAS-type mutations). However, invasive EFVPTC is associated with more *BRAF*-type mutations than noninvasive EFVPTC (which is associated with more *RAS*-type mutations), and follicular variants of papillary thyroid carcinoma are more frequently found to harbor *RAS*-type mutations than classic variants of papillary thyroid carcinoma (more *BRAF*-type mutations) [55].

The tumor invades the capsule and spreads into surrounding thyroid tissue or has lymphovascular invasion. Papillary thyroid cancer nuclear features include nuclear expansion, overlapping, and crowding, as well as elongation and irregularities in the nuclear membrane (e.g., uneven shapes, grooves, and pseudoinclusions, clearing with margination/glassy nuclei). Follicular growth patterns with copious colloids can be microfollicular, normofollicular, or macrofollicular.

### Oncocytic carcinoma of the thyroid

The new WHO criteria for ‘The Oncocytic Carcinoma of the Thyroid’ state that invasive malignant follicular cell neoplasms must consist of oncocytic cells with a cutoff value of at least 75%, and they must lack the nuclear characteristic of PTC as well as other high-grade characteristics. Hürthle described parafollicular C cell cancer, hence the name Hürthle cell carcinoma is now regarded to be a misnomer and has been replaced by parafollicular C cell cancer. Because of a significant buildup of defective mitochondria, oncocytic cells feature rich granular eosinophilic cytoplasm. The malignant analog of oncocytic adenoma is OCA [56].

The average age of diagnosis of Oncocytic carcinoma is around 60 years, in comparison to follicular carcinoma in which the average age of diagnosis is around 50 years [57]. OCA has a higher F: M ratio in comparison to follicular thyroid cancer (1.^6^:^1^). However, it is still lower than follicular thyroid cancer [58].

The clinical results of OCAs vary; therefore they are categorized as either minimally invasive (those with only capsular invasion), encapsulated angioinvasive, or broadly invasive. Tumors should be assessed for enhanced mitotic activity (three or more mitoses per ten high-power fields/2 mm^2^) and tumor necrosis to determine the likelihood of OCA progressing to oncocytic poorly differentiated thyroid carcinoma. Patient age, size of the tumor, extension beyond thyroid, blood vessel invasion, and the presence of distant metastases are all prognostic factors for OCA. The presence of distant metastases at the time of diagnosis is the most critical prognostic factor for OCA [59].

Oncocytic thyroid cancers, both benign and malignant, have been found to exhibit alterations in the mitochondrial DNA electron transport chain of complex I subunit genes [48].

## CONCLUSION

This 2022 updated volume of thyroid neoplasm incorporates both novel concepts vital to patient management. The new classification adds a new term: follicular nodular disease. Molecular analyses of individual nodules in such cases have revealed that a good proportion of goitrous nodules are monoclonal and represent neoplastic proliferations making it impossible to distinguish between non-neoplastic and benign neoplastic follicular neoplasms. The new category of low-risk neoplasm NIFTP, UMP, and HTT was added. The goal of the tumor terminology is to decrease the possibility of unnecessary treatment of low-risk neoplasms. The new classification method highlights the need for subtyping PTC based on histomorphologic indicators rather than tumor size. Similar to the traditional definition of ‘poorly differentiated thyroid carcinoma’ according to the Turin criteria, the newly defined ‘differentiated high-grade thyroid carcinoma’ encompasses PTCs, follicular thyroid carcinomas, and oncocytic carcinomas with high-grade characteristics linked to a worse prognosis. The squamous cell subtype of anaplastic thyroid cancer has also recently been characterized as a distinct morphologic pattern.

In the FTC, oncocytic carcinoma is defined as a population of oncocytic cells that exceeds 75%. The international medullary thyroid cancer grading scheme was designed for the two-tiered grading of medullary thyroid carcinoma, and it was independent of AJCC staging. We conclude that the 5^th^ edition of the WHO classification of thyroid tumor contains appropriate changes to nomenclature, grading, and prognostication of thyroid proliferations based on pathologic features and molecular profile. Therefore, it is important that endocrinologists and practicing physicians who manage thyroid nodules acquaint themselves with this new classification scheme.
